# Why and how to use the body's own stem cells for regeneration in musculoskeletal disorders: a primer

**DOI:** 10.1186/s13018-022-02918-8

**Published:** 2022-01-21

**Authors:** John P. Furia, Mark A. Lundeen, Jason L. Hurd, David A. Pearce, Christopher Alt, Eckhard U. Alt, Christoph Schmitz, Nicola Maffulli

**Affiliations:** 1 SUN Orthopedics of Evangelical Community Hospital, Lewisburg, PA USA; 2 Sanford Orthopedics & Sports Medicine Fargo, Fargo, ND USA; 3 Sanford Orthopedics & Sports Medicine Sioux Falls, Sioux Falls, SD USA; 4 Sanford Research, Sioux Falls, SD USA; 5 Sanford Health, Sioux Falls, SD USA; 6 Sanford School of Medicine, University of South Dakota, Sioux Falls, SD USA; 7 InGeneron, Inc., TX Houston, USA; 8 Institute of Anatomy, Faculty of Medicine, LMU Munich, Pettenkoferstr. 11, 80331 Munich, Germany; 9 IsarKlinikum, Munich, Germany; 10 Department of Musculoskeletal Disorders, Faculty of Medicine and Surgery, University of Salerno, Salerno, Italy; 11 Centre for Sports and Exercise Medicine, Barts and The London School of Medicine and Dentistry, Mile End Hospital, Queen Mary University of London, London, England; 12 School of Pharmacy and Bioengineering, Guy Hilton Research Centre, Keele University School of Medicine, Stoke on Trent, England

**Keywords:** Adipose-derived regenerative cells, ADRCs, Adipose-derived stem cells, ADSCs, Bone regeneration, Cartilage regeneration, Efficacy, Point of care treatment, Stem cell, Tendon healing without scar formation, Tendon regeneration, vaPS cells

## Abstract

**Background:**

Recently, the management of musculoskeletal disorders with the patients' own stem cells, isolated from the walls of small blood vessels, which can be found in great numbers in the adipose tissue, has received considerable attention. On the other hand, there are still misconceptions about these adipose-derived regenerative cells (ADRCs) that contain vascular-associated pluripotent stem cells (vaPS cells) in regenerative medicine.

**Methods:**

Based on our previous publications on this topic, we have developed a concept to describe the significance of the ADRCs/vaPS cells in the field of orthobiologics as briefly as possible and at the same time as precisely as possible.

**Results:**

The ADRCs/vaPS cells belong to the group of orthobiologics that are based on autologous cells. Because the latter can both stimulate a patient’s body's localized self-healing power and provide new cells that can integrate into the host tissue during the healing response when the localized self-healing power is exhausted, this group of orthobiologics appears more advantageous than cell-free orthobiologics and orthobiologics that are based on allogeneic cells. Within the group of orthobiologics that are based on autologous cells, enzymatically isolated, uncultured ADRCs/vaPS cells have several advantages over non-enzymatically isolated cells/microfragmented fat as well as over uncultured bone marrow aspirate concentrate and cultured cells (adipose-derived stem cells, bone marrow-derived mesenchymal stem cells).

**Conclusions:**

The use of ADRCs/vaPS cells can be seamlessly integrated into modern orthopedic treatment concepts, which can be understood as the optimization of a process which—albeit less efficiently—also takes place physiologically. Accordingly, this new safe and effective type of treatment is attractive in terms of holistic thinking and personalized medicine.

## Background

When talking about regenerative medicine in general and stem cells in particular, it is necessary to clarify at least some of the aspects of the physiology of these cells. Many lay people automatically think of embryonic stem cells when "stem cell therapy" is mentioned, but in reality currently no clinical application exists for embryonic stem cells. This is based not only on ethical concerns and the risk of development of teratomas (i.e. tumors derived from embryonic stem cells), but also on the allogeneic nature of embryonic cells [1, 2]. Even for so-called induced pluripotent stem cells (iPS cells), the development of which was honored with a Nobel Prize for Medicine in 2012, clinical applications are missing, not only for the complexity of the procedure, but particularly based on the risk of malignant transformation of these cells (i.e. the development of cancer) [1, 2]. For completeness, the transplantation of stem cells from the bone marrow in leukemia [3] should be mentioned, but this will not be discussed here further.

Under physiological conditions, maintenance and restoration of organ function is mostly achieved by local cells, including so-called tissue resident stem cells [4, 5]). However, in the event of acute trauma or disease, the sudden demand of new cells during the healing response may exceed the plasticity of the local cell populations. Furthermore, the ability of the tissue resident stem cells to re-enter the cell cycle and to asymmetrically divide is limited, which eventually limits the extent of self-renewal (and, thus, the self-healing power of the body) following major loss of cells in damaged tissue, such as during aging, after an infarction or with non-healing wounds etc.

On the other hand, there is a further type of stem cells present in the adult body, with the potential to develop (differentiate) into cells of all three embryonic germ layers (ectoderm, mesoderm, endoderm) [1, 2, 6]. These cells, which are termed vascular associated pluripotent stem cells (vaPS cells), are located in the walls of small blood vessels [1, 2]. Since blood vessels are the basis for the formation of tissue and organs in a developing body, these vaPS cells are also found in every organ of the adult body, including adipose tissue, heart, skin, bone marrow, skeletal muscle and tendons [1]. It is currently unknown to which extent these vaPS cells participate in the physiological maintenance and restoration of organ functions. In any case, unlike embryonic stem cells and iPS cells, the vaPS cells do not have their own, intrinsic program for the formation of new tissue, but become active in response to specific signals released and transmitted by diseased tissue [1]. Considering this fundamental difference, the vaPS cells have become an attractive option for regenerative therapy purposes without the risk of malignant transformation.

As long as the aforementioned local self-healing power of the body is sufficient to restore physiological body structures and functions in the event of trauma or disease, all treatment efforts should primarily focus on this. A variety of methods, including but not limited to physiotherapy, osteopathy, extracorporeal shock wave therapy (ESWT) [7], laser therapy [8] and the injection of platelet-rich plasma (PRP) [9], can make valuable contributions through stimulation of local regeneration.

However, a patient’s body's localized self-healing power can eventually exhaust. As a consequence, physiological body structures and functions can no longer be restored by the local stem cell pool. For example, in chronic wounds cells obtained during surgical wound debridement can no longer multiply to the extent that is necessary for adequate wound healing [10]. If a similar condition exists with damages in the musculoskeletal system, further conservative measures will have a high risk of failure. In essence, one can treat the patient with as much physiotherapy, ESWT, laser or other modalities as desired, and one can inject as much PRP as one wishes and patients request: these interventions will not work, or they only work to a limited extent because the cells that are supposed to effect the repair are simply not there any longer or cannot adequately react to stimulation.

**Fig. 1:**
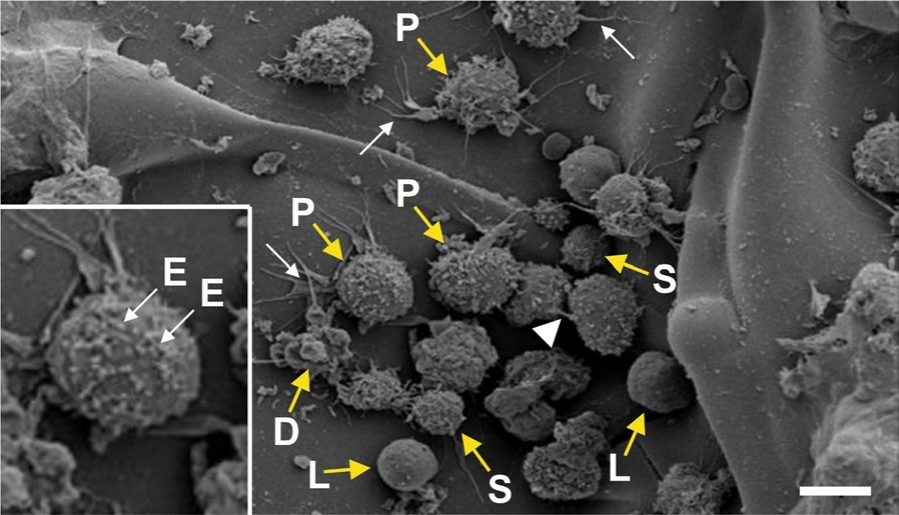
Scanning electron micrograph of ADRCs obtained from human abdominal adipose tissue (figure taken from [1] and with kind permission [12]). P, precursor cells; S, small cells; D, dying cells; L, lymphocytes; E, exosomes. The white arrows point to actin filaments and the white arrowhead to a microchannel between two cells. The bar corresponds to 10 µm (20 µm in the enlarged section)

This is exactly where the targeted use of the body's own vaPS cells comes into play, because they can be harvested and isolated from the body's own adipose tissue. Practically every one of us has a certain amount of body fat, which the organism can spare, and which can be obtained by mini-liposuction on the abdomen, the flanks or the thighs in an outpatient procedure with low risk and without general anesthesia; 100 g of adipose tissue are sufficient in most instances. Adipose derived regenerative cells (ADRCs) (which contain the vaPS cells [1]) can then be isolated from the adipose tissue using relatively simple technologies, whereby enzymatic processes are overall clearly superior to other, non-enzymatic processes [6]. It should be considered that some of the non-enzymatic processes may pose a risk that the ADRCs are not or not completely released from the surrounding connective tissue (e.g. [11]), limiting their effectiveness and increasing the risk of occlusions of small blood vessels if corresponding tissue fragments reach small blood vessels. Figure 1 shows ADRCs enzymatically isolated from human adipose tissue.

ADRCs are a mixture of cells including vaPS cells, progenitor cells, cells of the walls of the blood vessels (pericytes, endothelial cells, endothelial precursor cells and fibroblasts) and blood cells. Until a few years ago, it was thought that it was important to isolate the stem cells from the ADRCs in the next step and to multiply them in the laboratory (i.e. in cell culture) before using them therapeutically, resulting in so-called adipose derived stem cells (ADSCs). However, there is now good evidence that uncultured ADRCs are superior to cultured ADSCs for regeneration of tendons and bone [13, 14]. One of the reasons for this is that uncultured ADRCs contain cell types that can no longer be found in cultured ADSCs [15]. In addition, the conditions in the cell culture are at least not improving the function of the cells [16].

The use of fresh, uncultured ADRCs instead of cultured ADSCs has two other important advantages for the patient: (1) as the cells are not cultivated in a laboratory, the possible risk of contamination by bacteria and viruses is avoided, and (2) treatment with uncultured ADRCs is a real point of care procedure. Within a very short time span and in the same surgical setting, the adipose tissue can be obtained by mini-liposuction and the ADRCs can be injected to the point in the body where they are needed.

As evidenced by a large number of animal studies, treatment of pathologies of the musculoskeletal system with ADRCs is safe (i.e. does not lead to the development of cancer and other undesirable side effects) and treatment with ADRCs or ADSCs leads to a significant improvement of the structure and function of a damaged organ or tissue (e.g. [17–19]). Based on these highly positive results, treatment of human patients specifically with uncultured ADRCs started a few years ago. At this point, we would particularly like to address the treatment of symptomatic, partial-thickness rotator cuff tear (sPTRCT). Specifically, in a feasibility study approved by the U.S. Food and Drug Administration (FDA), we demonstrated for the first time that in patients suffering from sPTRCT who had not responded to over six weeks of conservative management (indicating that the self-healing power of the body had been exhausted), a single application of ADRCs led to rapid and long-lasting improvement in the clinical situation, with an improvement in the American Shoulder and Elbow Surgeons Standardized Shoulder Assessment Form (ASES) total score from 58.7 ± 19.2 (mean ± standard error of the mean) before treatment to 86.1 ± 4.9 at 24 weeks post treatment and 89.4 ± 4.9 one year post treatment [20] (the maximum ASES total score with complete freedom from pain and unrestricted mobility of the shoulder is 100). The results of a control group of patients treated with corticosteroid injections (a standard therapy for the condition at hand) were statistically significantly worse than the results of the patients treated with ADRCs (in the control group the mean ASES score was 50.6 ± 6.7 before treatment, 60.8 ± 6.2 at 24 weeks post treatment and 68.4 ± 4.4 one year post treatment [20]). In retrospect, the poor performance of the standard therapy (injection of corticosteroid) is not really surprising when it becomes clear that, when the local self-healing power of the body is exhausted, the injection of corticosteroids certainly leads to reduction of inflammation (and thus pain relief) in the affected shoulder, but cannot result in healing.

On the other hand, this study [20] could not answer the question of what exactly the ADRCs had done to the partial ruptures of the supraspinatus tendon. However, one co-author of this paper (E.A.) suffered from sPTRCT caused by a bicycle accident about five years ago and was treated with his own ADRCs. As part of a so-called medical self-test, for the first time worldwide a biopsy was taken from the partially ruptured supraspinatus tendon ten weeks after it had been treated with ADRCs. The biopsy then was examined in the laboratory of another co-author of this paper (C.S.) with a multitude of immunohistochemical markers [21]. We were able to demonstrate that regeneration without scar formation had taken place in the damaged tendon treated with ADRCs [22]. The special feature of this finding is that this type of regeneration had so far only been observed in fetal tendons [23].

In the meantime, a large number of patients suffering from various pathologies of the musculoskeletal system have been successfully treated with ADRCs both in individual healing attempts and controlled clinical trials, with sPTRCT, cartilage defects of the knee and osteoarthritis of the facet joints [1, 24–26] being the most important indications.

Of note, no special follow-up treatment is necessary after the application of ADRCs. Accordingly, patients can return to routine care immediately after the application of ADRCs.

In summary, the use of ADRCs in treatments of pathologies of the musculoskeletal system seamlessly fits into modern orthopedic treatment concepts. The patients receive treatment with their own body's self-healing power, which is just recovered and transferred from one “healthy” site to another site of the body in need for repair. This reflects a natural and intrinsically existing mechanism of the body, to mobilize stem cells from adipose tissue (however in often not sufficient amounts) and transfer cells for “self-healing” to damaged organs and tissue in need for repair [27].

The ADRCs/vaPS cells belong to the larger field of orthobiologics, which have recently been addressed in a number of comprehensive reviews (e.g. [28–30]) (Table 1).

**Table 1:** Overview on biologics according to the U.S. Food and Drug Administration

*Cell-free orthobiologics*	Examples
Platelet rich plasma	[31]
Exosomes	[32]
Amniotic fluid	[33]
*Orthobiologics that are based on allogeneic cells*	
Allogeneic mesenchymal stem cells (MSCs) derived from respectively placenta, umbilical cord or umbilical cord blood	[34–36]
Allogeneic bone marrow-derived MSCs (BM-MSCs)	[37]
Allogeneic adipose-derived stem cells (ADSCs)	[38]
*Orthobiologics that are based on autologous cells*	
Autologous ADRCs	[20]
Autologous ADSCs	[39]
Autologous, micro-fragmented fat (from liposuction)	[40]
Bone marrow aspirate concentrate	[41]
Autologous BM-MSCs	[42]
Chondrocyte transplants	[43]
Autologous, activated peripheral blood stem cells	[44]
*Other orthobiologics*	
Tissue-engineered patches	[45]
Cadaver grafts	[46]
Modulation of the immune system	[47]
*Other biologics with currently limited or no relevance in orthopedics*	
Recombinant therapeutic proteins	
Allergenics	
Vaccines	
Gene therapy	

According to the above, orthobiologics can generally be classified serving the following needs: (1) stimulating a patient’s body's localized self-healing power, and (2) providing new cells that can integrate into the host tissue during the healing response when the localized self-healing power is exhausted.

Of note, all cell-free orthobiologics as well as all orthobiologics that are based on allogeneic cells can in principle only address the former, because differentiated cells derived from allogeneic stem cells are recognized by the immune system of the host organism and removed [48]. Furthermore, repeated intra-articular injection of allogeneic stem cells in an equine model led to adverse responses, suggesting immune recognition of allogeneic stem cells upon a second exposure [49]. These effects were not observed after repeated intra-articular injection of autologous stem cells in the same model [49].

In contrast, orthobiologics that are based on autologous cells can address both the former and the latter, which makes them appear more advantageous than cell-free orthobiologics and orthobiologics that are based on allogeneic cells. It is obvious that this must be demonstrated in adequate, randomized controlled trials in the future (e.g. [50]).

Within the group of orthobiologics based on autologous cells, one has to differentiate between point of care procedures (e.g. uncultured ADRCs/vaPS cells and bone marrow aspirate concentrate (BMAC)) and procedures that include cell culturing (e.g. cultured ADSCs and bone marrow-derived mesenchymal stem cells). The disadvantages of the procedures that include cell culturing (selection of cells, exposing cells with the conditions of cell culture) are addressed above and explained in detail in the literature [2, 16]. The same applies to the advantages of ADRCs over BMAC (higher risk of opening bone marrow; orders of magnitude fewer stem cells in the same amount of BMAC compared with ADRCs) [2].

All mentioned procedures may be combined with tissue-engineered patches and/or cadaver grafts, which however requires surgical procedures to apply them. In addition, all cells that are derived from cell culture (including chondrocyte transplants) do not meet the criteria of ‘minimally manipulated’ as defined in U.S. Code of Federal Regulations 21 CFR 1271.10(a) (details and consequences are provided in [2]). The European Medicines Agency considers cells that are derived from cell culture as an Advanced Therapy Medicinal Product (ATMP) (details are also provided in [2]).

Finally, there remains one important question: why cannot the organism physiologically mobilize the vaPS cells in sufficient quantity from a distant reservoir in the walls of small blood vessels and bring them to sites where the self-healing power is exhausted? One possible answer is that degenerative processes of tissues of the musculoskeletal system (if they were not induced, for example, by sports accidents, etc.) in the vast majority of cases do not appear until an age that has never been subject to optimization pressure in evolution. In this regard, our concept to "give nature a helping hand" to optimize an intrinsic process that naturally exists (albeit at a less sufficient level) is safe and efficient and thus appears logical and recommendable.

## Data Availability

The data that support the findings of this study are available from the corresponding author.

## Abbreviations

ADRCs: Adipose derived regenerative cells
ADSCs: Adipose derived stem cells
ASES: American Shoulder and Elbow Surgeons Standardized Shoulder Assessment Form
BMAC: Bone marrow aspirate concentrate
ESWT: Extracorporeal shock wave therapy
FDA: U.S. Food and Drug Administration
iPS cells: Induced pluripotent stem cells
vaPS cells: Vascular associated pluripotent stem cells
PRP: Platelet-rich plasma
sPTRCT: Symptomatic, partial-thickness rotator cuff tear
